# Supplementary material for: A proteome-wide quantitative platform for nanoscale spatially resolved extraction of membrane proteins into native nanodiscs
Source: Nat Methods. 2024 Nov 28;22(2):412–21. doi: 10.1038/s41592-024-02517-x (PMC11810782; doi:10.1038/s41592-024-02517-x)
Supplement: Supplementary file 1 — Supplementary Tables 1–3, Supplementary WebApp guide and References. [file 41592_2024_2517_MOESM1_ESM.pdf]

nature methods

Supplementary information

https://doi.org/10.1038/s41592-024-02517-x

A proteome-wide quantitative platform for nanoscale spatially resolved extraction of membrane proteins into native nanodiscs

In the format provided by the authors and unedited

Supplementary Table 1: List of high-resolution structures of membrane proteins solved in membrane active polymers. The ones purified from eukaryotic cells are highlighted in red

Protein	Polymer	Year	Purified From	Resolution	Reference
ACRB	SMA 2:1	2018	E. coli	8.8 Å	[1]
ACIII	SMA 3:1	2018	F. johnsoniae	3.4 Å	[2]
ZipA:FtsZ	SMA 2:1	2019	E. coli	16 Å	[3]
KimA	SMA 2:1	2020	E. coli	3.7 Å	[4]
hTRPM4	AASTY-B	2020	HEK293	18 Å	[5]
BdSLAC1	SMA (not specified which type)	2021	S. pombe	2.97 Å	[6]
Cyt bo_3	SMA 3:1	2021	E. coli	2.55 Å	[7]
ELIC	SMA 3:1	2021	E. coli	2.5 Å	[8]
Yna1	SMA 2:1	2021	E. coli	2.4 Å	[9]
GlyR	SMA2:1	2021	Sf9	3.2 Å	[10]
Bam complex	SMA 2:1	2022	E. coli	3.6 Å	[11]
cASIC1	SMA 2:1	2020	HEK293S GnTI	2.8 Å	[12]
HIV-1 Env	SMA 2:1	2023	A549	4.1 Å	[13]
WbaP	SMA 2:1	2023	E. coli	Not specified	[14]
Cyt bc_1	SMA (not specified which type)	2023	R. sphaeroides	2.9 Å	[15]
GPIb-1X-V	SMA 3:1	2023	Expi293F	11 Å	[16]
integrin αIIbβ3	NCMNP7b	2023	platelets	3.7 Å	[17]

Supplementary Information Table 2: Polymer description

Polymer Name	Description	Synthesis Methods	Origin
SMA140	Styrene, Maleic Acid ratio 1:1; MW 6.5 KDa	CSTR	Commercial
SMA200	Styrene, Maleic Acid ratio 2:1; MW 6.5 KDa	CSTR	Commercial
SMA300	Styrene, Maleic Acid ratio 3:1; MW 6.5 KDa	CSTR	Commercial
AASTY645	Styrene, Acrylic Acid ratio 55:45; MW 6 KDa	RAFT	Commercial
AASTY650	Styrene, Acrylic Acid ratio 50:50; MW 6 KDa	RAFT	Commercial
AASTY655	Styrene, Acrylic Acid ratio 45:55; MW 6 KDa	RAFT	Commercial
AASTY1145	Styrene, Acrylic Acid ratio 55:45; MW 11 KDa	RAFT	Commercial
AASTY1150	Styrene, Acrylic Acid ratio 50:50; MW 11 KDa	RAFT	Commercial
AASTY1155	Styrene, Acrylic Acid ratio 45:55; MW 11 KDa	RAFT	Commercial
CS20	Chloro Styrene, Maleic Acid ratio 1:1; MW 6.0 KDa	RAFT	Home made
CS40	Chloro Styrene, Maleic Acid ratio 1:1; MW 6.6 KDa	RAFT	Home made
CS60	Chloro Styrene, Maleic Acid ratio 1:1; MW 7.7 KDa	RAFT	Home made
CS80	Chloro Styrene, Maleic Acid ratio 1:1; MW 8.8 KDa	RAFT	Home made
AASTY80	Styrene, Acrylic Acid ratio 55:45; MW 5.1 KDa	RAFT	Home made

Supplementary Information Table 3: Extraction conditions

Extraction Condition	Buffer Condition	Salt Concentration	10% glycerol	% polymer
SMA140	20 mM HEPES pH 7.5	100mM NaCl	N	1%
SMA200	50 mM TrisHCl pH 8.1	300mM NaCl	Y	1%
SMA300	20 mM HEPES pH 7.5	100mM NaCl	N	1%
AASTY645	20 mM HEPES pH 7.5	100mM NaCl	N	1%
AASTY650	20 mM HEPES pH 7.5	100mM NaCl	N	1%
AASTY655	20 mM HEPES pH 7.5	100mM NaCl	N	1%
AASTY1145	20 mM HEPES pH 7.5	100mM NaCl	N	1%
AASTY1150	20 mM HEPES pH 7.5	100mM NaCl	N	1%
AASTY1155	20 mM HEPES pH 7.5	100mM NaCl	N	1%
CS20	50 mM TrisHCl pH 8.1	150 mM NaCl	Y	1.5%
CS40	50 mM TrisHCl pH 8.1	150 mM NaCl	Y	1.5%
CS60	50 mM TrisHCl pH 8.1	150 mM NaCl	Y	1.5%
CS80	50 mM TrisHCl pH 8.1	150 mM NaCl	Y	1.5%
AASTY80	50 mM TrisHCl pH 7.8	150 mM NaCl	Y	1.5%

Other Supplementary Information: WebApp Guide

The repository can be accessed on any web-connected browser from desktop or mobile devices. As this is a web-based platform no installation is necessary to perform searches. To search the open-source database go to: www.polymerscreen.yale.edu. This will take you to the landing page:

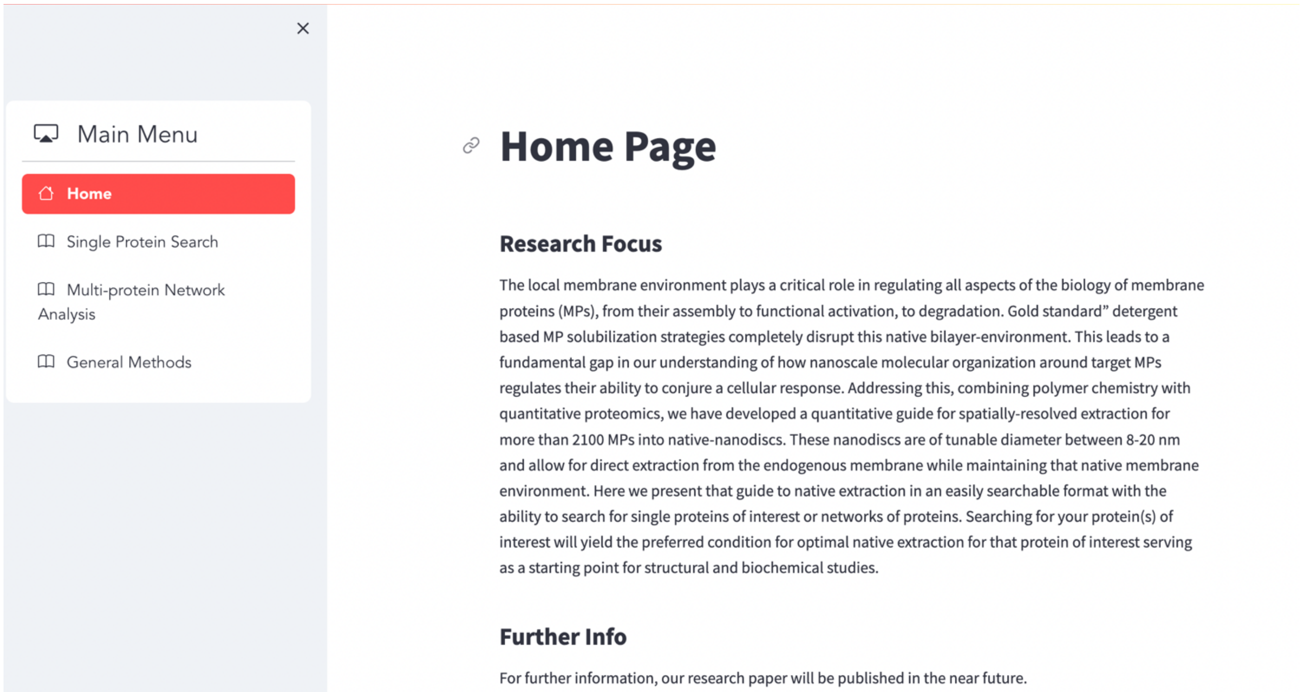

To explore solubilization efficiency for a single protein, click on "Single Protein Search":

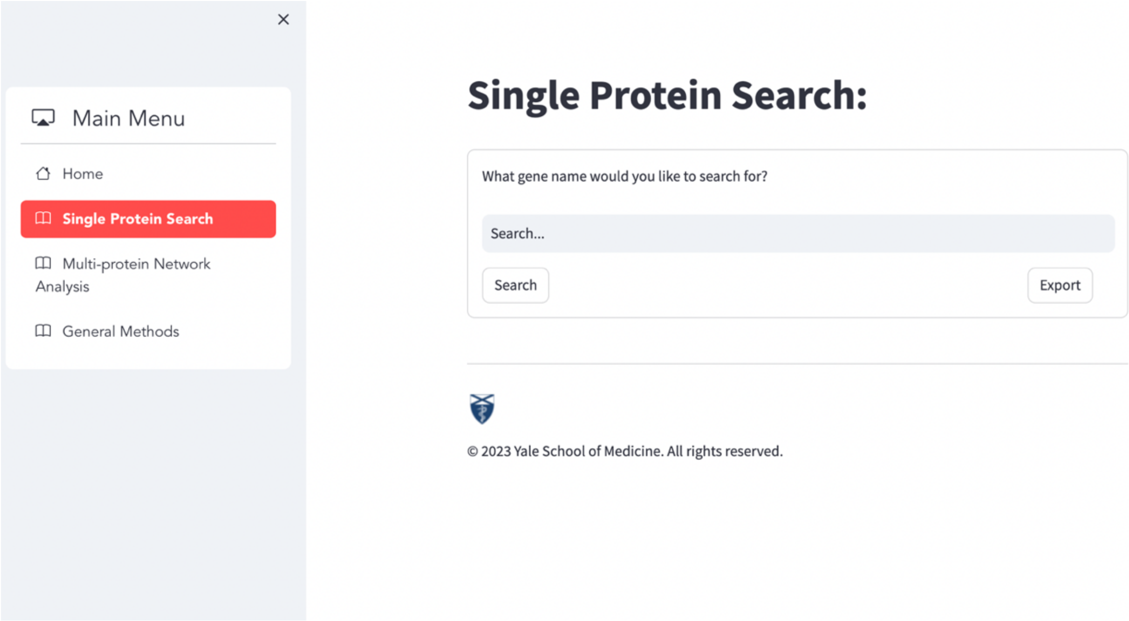

Enter the Gene Name of the protein you are interested in searching for. The webpage will return the search results in the form of a bar graph where 100% indicates the optimal polymer for extraction of this protein (in the case shown below AASTY650). You also have the option to export the quantitative data into an excel spread sheet using the “Export” button:

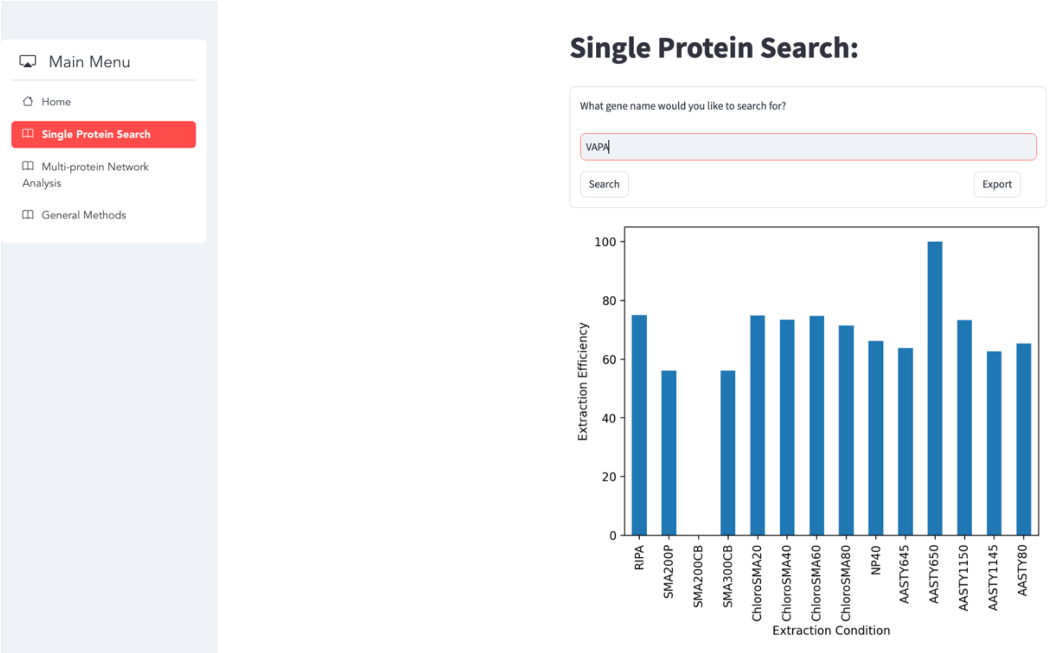

To explore solubilization efficiency for multiple proteins, or a protein complex, click on “Multi-protein Network Analysis”:

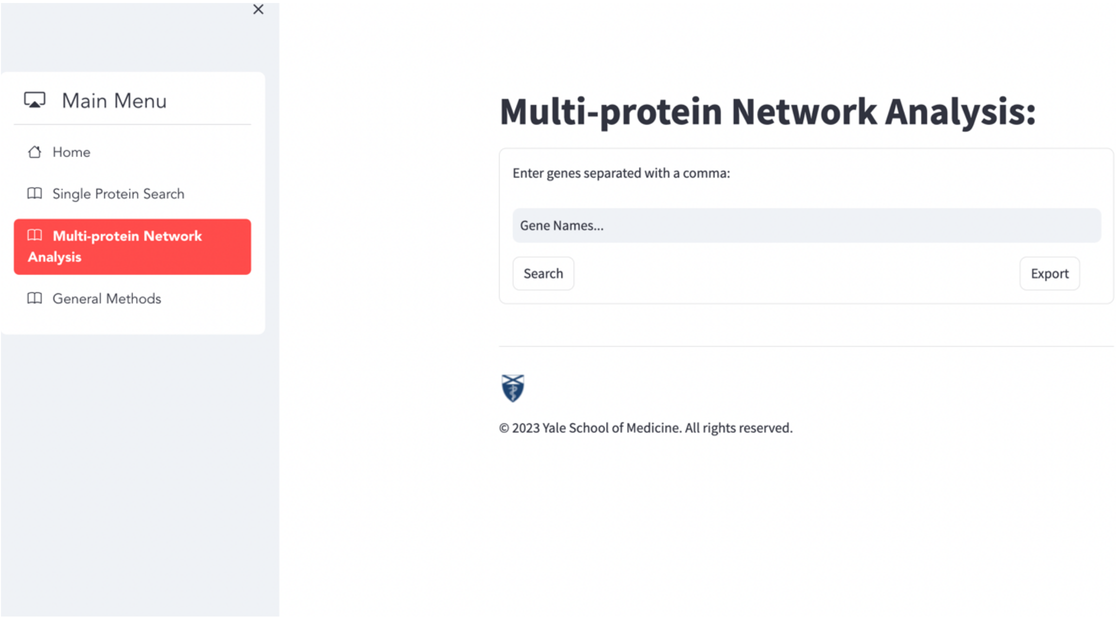

Enter the gene name of every protein in your network of interest separated by a comma and a space. The webpage will return a bar graph with the calculated “Solubilization Index” where the bar scaled closest to 1 represents the optimal solubilization condition to extract the protein network of interest (in the example below SMA200). The quantitative data can be exported to an excel spreadsheet using the “Export” button:

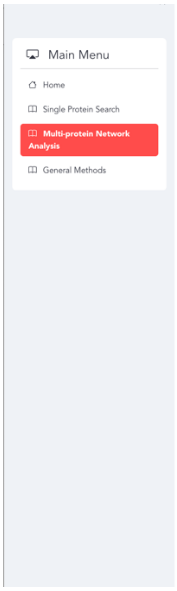

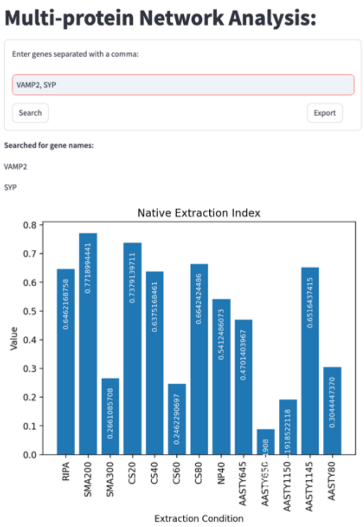

Supplementary Information References:

1. Parmar, M. *et al.* Using a SMALP platform to determine a sub-nm single particle cryo-EM membrane protein structure. *Biochim. Biophys. Acta Biomembr.* **1860**, 378–383 (2018).

2. Sun, C. *et al.* Structure of the alternative complex III in a supercomplex with cytochrome oxidase. *Nature* **557**, 123–126 (2018).

3. Lee, S. C. *et al.* Nano-encapsulated Escherichia coli Divisome Anchor ZipA, and in Complex with FtsZ. *Sci. Rep.* **9**, 18712 (2019).

4. Tascón, I. *et al.* Structural basis of proton-coupled potassium transport in the KUP family. *Nat. Commun.* **11**, 626 (2020).

5. Smith, A. A. A. *et al.* Lipid nanodiscs via ordered copolymers. *Chem* (2020) doi:10.1016/j.chempr.2020.08.004.

6. Deng, Y.-N. *et al.* Structure and activity of SLAC1 channels for stomatal signaling in leaves. *Proc Natl Acad Sci USA* **118**, (2021).

7. Li, J. *et al.* Cryo-EM structures of Escherichia coli cytochrome bo3 reveal bound phospholipids and ubiquinone-8 in a dynamic substrate binding site. *Proc Natl Acad Sci USA* **118**, (2021).

8. Kumar, P., Cymes, G. D. & Grosman, C. Structure and function at the lipid-protein interface of a pentameric ligand-gated ion channel. *Proc Natl Acad Sci USA* **118**, (2021).

9. Catalano, C. *et al.* Cryo-EM Structure of Mechanosensitive Channel YnaI Using SMA2000: Challenges and Opportunities. *Membranes (Basel)* **11**, (2021).

10. Yu, J. *et al.* Mechanism of gating and partial agonist action in the glycine receptor. *Cell* **184**, 957-968.e21 (2021).

11. Doyle, M. T. *et al.* Cryo-EM structures reveal multiple stages of bacterial outer membrane protein folding. *Cell* **185**, 1143-1156.e13 (2022).

12. Yoder, N. & Gouaux, E. The His-Gly motif of acid-sensing ion channels resides in a reentrant "loop" implicated in gating and ion selectivity. *eLife* **9**, (2020).

13. Wang, K. *et al.* Asymmetric conformations of cleaved HIV-1 envelope glycoprotein trimers in styrene-maleic acid lipid nanoparticles. *Commun. Biol.* **6**, 535 (2023).

14. Dodge, G. J. *et al.* Mapping the architecture of the initiating phosphoglycosyl transferase from S. enterica O-antigen biosynthesis in a liponanoparticle. *BioRxiv* (2023) doi:10.1101/2023.06.16.545297.

15. Swainsbury, D. J. K. *et al.* Cryo-EM structure of the four-subunit Rhodobacter sphaeroides cytochrome bc1 complex in styrene maleic acid nanodiscs. *Proc Natl Acad Sci USA* **120**, e2217922120 (2023).

16. Lu, J. *et al.* Stoichiometry and architecture of the platelet membrane complex glycoprotein Ib-IX-V. *Biol. Chem.* (2023) doi:10.1515/hsz-2022-0227.

17. Adair, B. D., Xiong, J.-P., Yeager, M. & Arnaout, M. A. Cryo-EM structures of full-length integrin αIIbβ3 in native lipids. *Nat. Commun.* **14**, 4168 (2023).
