# Supplementary material for: A proteome-wide quantitative platform for nanoscale spatially resolved extraction of membrane proteins into native nanodiscs
Source: Nat Methods. 2024 Nov 28;22(2):412–21. doi: 10.1038/s41592-024-02517-x (PMC11810782; doi:10.1038/s41592-024-02517-x)
Supplement: Supplementary file 2 — Reporting Summary [file 41592_2024_2517_MOESM2_ESM.pdf]

nature portfolio

Corresponding author(s): Kallol Gupta

Last updated by author(s): 2024/09/26

Reporting Summary

Nature Portfolio wishes to improve the reproducibility of the work that we publish. This form provides structure for consistency and transparency in reporting. For further information on Nature Portfolio policies, see our Editorial Policies and the Editorial Policy Checklist.

Statistics

For all statistical analyses, confirm that the following items are present in the figure legend, table legend, main text, or Methods section.

n/a	Confirmed	
☐	☒	The exact sample size (*n*) for each experimental group/condition, given as a discrete number and unit of measurement
☐	☒	A statement on whether measurements were taken from distinct samples or whether the same sample was measured repeatedly
☒	☐	The statistical test(s) used AND whether they are one- or two-sided<br>*Only common tests should be described solely by name; describe more complex techniques in the Methods section.*
☒	☐	A description of all covariates tested
☒	☐	A description of any assumptions or corrections, such as tests of normality and adjustment for multiple comparisons
☐	☒	A full description of the statistical parameters including central tendency (e.g. means) or other basic estimates (e.g. regression coefficient) AND variation (e.g. standard deviation) or associated estimates of uncertainty (e.g. confidence intervals)
☒	☐	For null hypothesis testing, the test statistic (e.g. *F*, *t*, *r*) with confidence intervals, effect sizes, degrees of freedom and *P* value noted<br>*Give P values as exact values whenever suitable.*
☒	☐	For Bayesian analysis, information on the choice of priors and Markov chain Monte Carlo settings
☒	☐	For hierarchical and complex designs, identification of the appropriate level for tests and full reporting of outcomes
☒	☐	Estimates of effect sizes (e.g. Cohen's *d*, Pearson's *r*), indicating how they were calculated

Our web collection on statistics for biologists contains articles on many of the points above.

Software and code

Policy information about availability of computer code

Data collection	No software was used.
Data analysis	MaxQuant version 2.3.1.0 (RRID: SCR_014485) was used to ID peptides and perform label free quantitation (PXD052184). All size exclusion chromatography data was processed by using Origin 2022 software (RRID:SCR_014212). The database described in the manuscript is available at https://polymerscreen.yale.edu OR https://polymerscreen.streamlit.app. Additionally, all code is available in Zenodo at https://doi.org/10.5281/zenodo.13380404.

For manuscripts utilizing custom algorithms or software that are central to the research but not yet described in published literature, software must be made available to editors and reviewers. We strongly encourage code deposition in a community repository (e.g. GitHub). See the Nature Portfolio guidelines for submitting code & software for further information.

Data

Policy information about availability of data

All manuscripts must include a data availability statement. This statement should provide the following information, where applicable:

- Accession codes, unique identifiers, or web links for publicly available datasets
- A description of any restrictions on data availability
- For clinical datasets or third party data, please ensure that the statement adheres to our policy

All proteomics data is available via the PRIDE data repository at PXD052184. All datasets and codes have been deposited in zenodo https://doi.org/10.5281/zenodo.1338040439. All other data are included in the manuscript and/or supporting information.

Research involving human participants, their data, or biological material

Policy information about studies with human participants or human data. See also policy information about sex, gender (identity/presentation), and sexual orientation and race, ethnicity and racism.

Reporting on sex and gender	N/A
Reporting on race, ethnicity, or other socially relevant groupings	N/A
Population characteristics	N/A
Recruitment	N/A
Ethics oversight	N/A

Note that full information on the approval of the study protocol must also be provided in the manuscript.

Field-specific reporting

Please select the one below that is the best fit for your research. If you are not sure, read the appropriate sections before making your selection.

☒ Life sciences ☐ Behavioural & social sciences ☐ Ecological, evolutionary & environmental sciences

For a reference copy of the document with all sections, see nature.com/documents/nr-reporting-summary-flat.pdf

Life sciences study design

All studies must disclose on these points even when the disclosure is negative.

Sample size	All proteomics data was replicated across 4 technical replicates and 2 biological replicates for a total sample size of 8 replicates per data point. These sample sizes were chosen based off of standards in the field of proteomics where typically at least 2 biological replicates are required with at least 3 technical replicates. We believe that the 8 replicates we have per sample sufficiently capture both technical and biological variability within the samples.
Data exclusions	No data was excluded from this study.
Replication	All proteomics data was replicated across 4 technical replicates and 2 biological replicates for a total sample size of 8 replicates per data point. Agreement between biological replicates was good in all cases.
Randomization	Randomization was used in running the proteomics samples to ensure that all samples were subject to similar column conditions and to account for variability that could be introduced through column wear.
Blinding	Blinding was not used in this study. In order to conduct solubilization, researchers needed to know which polymers were being used.

Reporting for specific materials, systems and methods

We require information from authors about some types of materials, experimental systems and methods used in many studies. Here, indicate whether each material, system or method listed is relevant to your study. If you are not sure if a list item applies to your research, read the appropriate section before selecting a response.

Materials & experimental systems

n/a	Involved in the study	
☐	☒	Antibodies
☐	☒	Eukaryotic cell lines
☒	☐	Palaeontology and archaeology
☒	☐	Animals and other organisms
☒	☐	Clinical data
☒	☐	Dual use research of concern
☒	☐	Plants

Methods

n/a	Involved in the study	
☒	☐	ChIP-seq
☒	☐	Flow cytometry
☒	☐	MRI-based neuroimaging

Antibodies

Antibodies used

Primary Antibody: monoclonal rabbit Na/K ATPase (Abcam Cat# ab167390, 1:5000), monoclonal Synaptophysin Rabbit mAb (ABClonal Cat#A19122, 1:5000), monoconal VAMP2 Rabbit mAb (ABClonal Cat#A4235, 1:5000); Secondary Antibody: HRP Goat Anti-Rabbit IgG (H+L) (ABClonal Cat#AS014, 1:10000) clonality not specified.

Validation

Per the manufacturers websites, ab167390 was validated with WB, IC/ICC, flow cyt, and IHC-P A19122 was validated with WB, IC/ICC, and ELISA. A4235 was validated with WB, IF/ICC, and ELISA. AS014 was validated with WB, IHC-P, and ELISA.

Eukaryotic cell lines

Policy information about cell lines and Sex and Gender in Research

Cell line source(s)

Standard Hek293 and HeLa cell lines were used throughout this study. HEK293 cells can be purchased through ATCC Cat# CRL-1573. HeLa cells can be purchased through ATCC Cat# CRM-CCL-2.

Authentication

Cells were not authenticated.

Mycoplasma contamination

Cells were not tested for mycoplasm.

Commonly misidentified lines
(See ICLAC register)

No commonly misidentified cell lines were used in this study.

Plants

Seed stocks

Report on the source of all seed stocks or other plant material used. If applicable, state the seed stock centre and catalogue number. If plant specimens were collected from the field, describe the collection location, date and sampling procedures.

Novel plant genotypes

Describe the methods by which all novel plant genotypes were produced. This includes those generated by transgenic approaches, gene editing, chemical/radiation-based mutagenesis and hybridization. For transgenic lines, describe the transformation method, the number of independent lines analyzed and the generation upon which experiments were performed. For gene-edited lines, describe the editor used, the endogenous sequence targeted for editing, the targeting guide RNA sequence (if applicable) and how the editor was applied.

Authentication

Describe any authentication procedures for each seed stock used or novel genotype generated. Describe any experiments used to assess the effect of a mutation and, where applicable, how potential secondary effects (e.g. second site T-DNA insertions, mosiacism, off-target gene editing) were examined.
